# Fundamental workings of chemical substitution at the A-site of perovskite oxides— a ^207^Pb NMR study of Ba-substituted PbZrO_3_†

**DOI:** 10.1039/d2dt01302a

**Published:** 2022-11-09

**Authors:** Sonja Egert, Jurij Koruza, Hergen Breitzke, Changhao Zhao, Barbara Malič, Gerd Buntkowsky, Pedro B. Groszewicz

**Affiliations:** Eduard Zintl Institute for Inorganic and Physical Chemistry, Technical University of Darmstadt Darmstadt 64287 Germany gerd.buntkowsky@chemie.tu-darmstadt.de; Institute for Chemistry and Technology of Materials, Graz University of Technology Graz 8010 Austria; Department of Materials and Earth Sciences, Nonmetallic Inorganic Materials, Technical University of Darmstadt Darmstadt 64287 Germany; Electronic Ceramics Department, Jožef Stefan Institute Ljubljana Slovenia; Department of Radiation Science and Technology, Delft University of Technology Delft 2629JB Netherlands p.groszewicz@tudelft.nl

## Abstract

Lead zirconate (PbZrO_3_, PZ) is a prototype antiferroelectric (AFE) oxide from which state-of-the-art energy storage materials are derived by chemical substitutions. A thorough understanding of the structure–property relationships of PZ-based materials is essential for both performance improvement and the design of more environmentally friendly replacements. (Pb_1−*x*_Ba_*x*_)ZrO_3_ (PBZ) can serve as a model system for studying the effect of A-site substitution in the perovskite lattice, with barium destabilizing the AFE state. Here, the two-dimensional ^207^Pb solid-state NMR spectra of PZ and PBZ were recorded to analyze the local structural role of barium substitution. At low substitution levels, ^207^Pb NMR spectroscopy reveals the presence of Pb–O bond length disorder. Upon crossing the threshold value of *x* for the macroscopic phase transition into a ferroelectric (FE) state, the barium cations cause local-scale lattice expansions in their vicinity, resulting in the collapse of two lead lattice sites into one. The stabilization of the larger volume site coincides with the favoring of larger lead displacements. We also observed more covalent bonding environments which may originate from the lower polarizability of the barium cations, facilitating the formation of stronger Pb–O bonds in their vicinity. From the local structural point of view, we propose that the substitution-induced AFE → FE phase transition is therefore related to an increasing correlation of larger lead displacements in larger oxygen cavities as the barium content increases. Our results also highlight ^207^Pb NMR spectroscopy as a valuable method for the characterization of the structure–property relationships of PbZrO_3_-based AFE and FE oxides.

## Abstract 2

^207^Pb NMR spectroscopy reveals the fundamental workings of chemical substitution at the A-site of perovskite oxides, and discloses how Ba^2+^ acts as the driving force of the antiferroelectric-to-ferroelectric phase transition in PbZrO_3_-based oxides.

## Graphical abstract

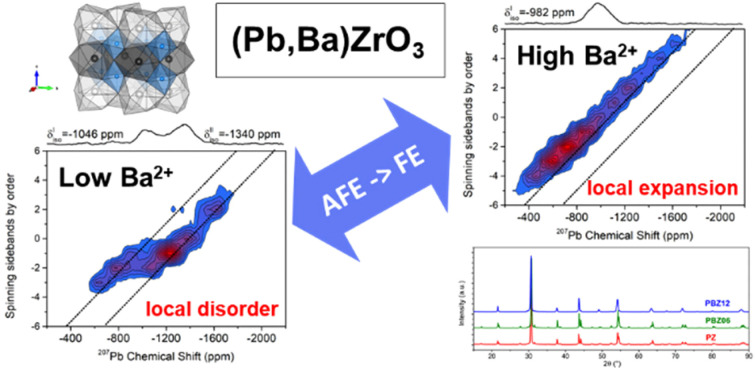

## Introduction

Antiferroelectric (AFE) oxides constitute a promising class of functional materials suitable for energy-storage applications^1^ or ferroic cooling.^2,3^ Among this class, compositions based on lead zirconate (PbZrO_3_, PZ) are considered to be state-of-the-art compositions. PZ was the first material for which antiferroelectricity was described^4–6^ and is arguably the best-studied AFE perovskite.^7,8^ It exhibits an orthorhombic symmetry with the space group *Pbam* below the Curie temperature *T*_C_ = 230 °C.^9,10^ Antiparallel displacements of lead cations,^11,12^ as well as long-range ordered *a*^−^*a*^−^*c*^0^ rotations of oxygen octahedra,^8,13^ are characteristic of its room temperature phase. Despite the long history of PZ, its structure and properties are far from being fully understood, with previous publications highlighting the complexity of the phase transition to the ferroelectric (FE) state.^14–17^ An intermediate phase^4^ is observable only under specific conditions.^18^ In addition, the question of disordered lead displacements even in the ground state has been raised.^19^

For practical applications of bulk ceramic AFEs, the AFE → FE phase transition must be both inducible by an electric field and reversible. While PZ is oftentimes regarded as a prototype AFE material, its field-induced phase transition is indeed only observable for single crystals^20^ or at elevated temperatures.^5^ This is because under ambient conditions the forward-switching field *E*_AF_ necessary to induce the transition typically exceeds the breakdown field of the ceramic.^1,10^ Since the 1950s, efforts have been made to lower *E*_AF_ by chemical substitution at both the A- and B-sites of the perovskite lattice.^10,21^

The effects of substituting Pb^2+^ with larger isovalent Ba^2+^ ions in (Pb_1−*x*_Ba_*x*_)ZrO_3_ (PBZ) solid solutions have previously been reviewed in detail.^8,21^ While *E*_AF_ is indeed lowered,^6,22^ the AFE phase is destabilized at the same time, causing the appearance of an intermediate, rhombohedral FE phase with a polar 〈111〉 axis at elevated temperatures.^6,22,23^ The field-induced transition between the two phases is then accompanied by an increase in lattice volume.^24^ It has been proposed that at *x* > 0.175, the FE phase is stabilized at room temperature,^25,26^ and relaxor behavior has been reported for *x* > 0.3.^25,27,28^ The destabilizing effect of barium has been ascribed to the larger ions inducing parallel displacements in the structure, thus increasing the symmetry and driving the transition from AFE to FE.^22^ It was also suggested that Ba^2+^ ions elongate the Zr–O distances in their local surroundings, resulting in an instability of the Zr^4+^ ions and the formation of permanent electric dipoles.^25^ In addition, evidence of partial disorder of the Pb^2+^ displacements was found in PBZ above 130 K.^25^

Environmental concerns about PZ-based functional ceramics have motivated the search for lead-free replacements.^29^ A thorough understanding of the structure–property relationships could contribute to smart material design, but established compositions such as (Pb,La)(Zr,Sn,Ti)O_3_ are complex. The study of PBZ as a simpler model system in comparison with the parent structure of PZ can give valuable insights into the isolated effect of A-site modification under the influence of a destabilizing ion. In a similar fashion, the structures of pure^30^ and modified^31^ NaNbO_3_ are of interest for the development of functional, lead-free ceramics.

With solid-state nuclear magnetic resonance (NMR) spectroscopy, structural information on a local scale can be obtained, including, for example, bond lengths or the distortion of local environments.^31,32^ This is valuable especially for disordered materials such as solid solutions and chemically substituted oxides. Due to the sensitivity of NMR spectroscopy to the local structure, in particular, this technique is able to provide insights into the immediate surroundings of cations in the lattice under the influence of chemical substitution, which can be supplemented with XRD and electrical measurements in order to include the average lattice structure and the macroscopic properties in a more complete picture of the material. While diffraction methods can only give averaged information in this case, NMR spectroscopy often provides insights into the presence of different local environments or parameter distributions.

Due to its high chemical shift (CS) dispersion, ^207^Pb NMR is specifically sensitive to changes in the chemical environment of lead cations. An overview of ^207^Pb NMR studies can be found in the review by Dybowski and Neue.^38^ As the CS interaction is typically the dominant influence in ^207^Pb spectra, empirical correlations between the structural and CS parameters are established for a series of lead-containing compounds.^34–37,40^ This makes it a suitable probe for the local environment of Pb and the effects of neighboring cation substituents on it, which play an essential role in the functional properties of PZ-based materials. Accessibility of this information has previously been demonstrated for PbZrO_3_-based perovskites.^33–36^ One of the challenges of the method lies in the high electron number of lead, which accounts for large polarizabilities and causes even small deviations of the electron cloud from spherical symmetry to result in large chemical shift anisotropies (CSA).^37^ Hence, broad line shapes or complicated spinning sideband (SSB) patterns under magic angle spinning (MAS) are characteristic aspects.^38^ Furthermore, the sensitivity of the chemical shift to the probe temperature is to be expected,^39^ and the spin–lattice relaxation times for known lead perovskite oxides have been found to vary in a broad range between 1 s and 160 s.^35^

While the effects of barium substitution on the global structure and phase transition temperatures of PbZrO_3_ are reported in previous literature, rationalization of the chemical and structural reasons for this effect remained elusive. This fact has motivated our current search for a local structural origin of the changes brought about by barium, particularly in relation to the chemistry of this substituent in perovskite oxides. In this work, we analyzed the ^207^Pb NMR spectra of pure PZ, (Pb_0.94_Ba_0.06_)ZrO_3_ (PBZ06), and (Pb_0.88_Ba_0.12_)ZrO_3_ (PBZ12) in order to determine how the local structure of Pb^2+^ responds to the modification with Ba^2+^. These experiments are unique in that they reveal considerable local structural disorder upon chemical substitution, which is interpreted in terms of the distribution of the shortest Pb–O bond lengths. Furthermore, our results indicate an increased Pb–O bond covalency in the FE structure, attributed to the lower polarizability of barium cations, thus providing experimental evidence for the mechanism which leads to the AFE destabilization caused by Ba^2+^. The large CSA prevented the determination of NMR parameters at moderate MAS rates, which are necessary for spin sintered ceramic sample pieces. This experimental challenge has been overcome by employing the 2D-PASS (Two-Dimensional Phase-Adjusted Spinning Sidebands) method.^41^ Here, we used it to separate overlapping SSB patterns from different lead sites and to obtain purely isotropic spectra at 8 kHz MAS. In addition, the CSA patterns of two different Pb^2+^ sites were extracted.

## Experimental

PbZrO_3_ (PZ), Pb_0.94_Ba_0.06_ZrO_3_ (PBZ06), and Pb_0.88_Ba_0.12_ZrO_3_ (PBZ12) were prepared by solid state synthesis. The starting powders PbO (Sigma, 99.9% purity), ZrO_2_ (TZ-0, Tosoh), and BaCO_3_ (Alfa, 99.8% purity) were mixed in a stoichiometric ratio using a planetary mill and calcined twice at 850 °C for 2 h with intermediate milling. The synthesized powders were pressed into pellets using uniaxial and isostatic pressure and subsequently sintered at 1250 °C for 2 h. The sintered samples were ground, annealed, and for electrical measurements electroded with gold. *ε*(*T*) and polarization–electric field hysteresis loops were determined using an HP 4192A impedance analyzer and an AixACCT TF2000 ferroelectric measurement setup, respectively. Before XRD analysis, the ground samples were annealed at 400 °C for 2 h. X-ray powder diffractograms were then recorded by using a Bruker D8 Advance diffractometer with CuK_α_ radiation. The analysis of the full patterns was performed using the Jana2006 package.^42^ The ^207^Pb solid-state NMR spectra were recorded using a Bruker Avance III spectrometer operating with a 7.1 T magnet. The sintered ceramic pellets were cut to dimensions of approximately 1.2 × 3 × 6.2 mm, packed in 4 mm zirconia rotors with γ-Al_2_O_3_ as a packing powder, and spun at the magic angle at a rate of 8 kHz. In addition, a small piece of sintered PBZ06 was crushed and annealed at 400 °C for 2 h to alleviate mechanical stresses and then the NMR spectra were recorded at 8 kHz MAS. ^207^Pb 2D-PASS experiments were carried out with five π pulses of 5.8 μs following the initial π/2 pulse of 2.6 μs, and a relaxation delay of 10 s. A modified version of the sequence with a shifted echo was used to accommodate for the free induction decays of PBZ dying out in less than 150 μs,^40^ with a duration of one rotor period added to the delay before and after the last π pulse. Sixteen pitch increments were used to separate the spinning sidebands by following the timings given by Antzutkin *et al.*^41^ The phase of each of the π pulses was cycled independently in 120° steps, yielding the 243-step scheme given in therein. 972 or 1215 scans were averaged on each pitch. ^207^Pb chemical shifts were referenced to the Pb^II^ signal of commercial PbZrO_3_ at −1363 ppm following the data published by Zhao *et al.*^35^ Line shape simulations of CSA patterns were carried out with the program DMFit.^43^

## Results & discussion

**Fig. 1:**
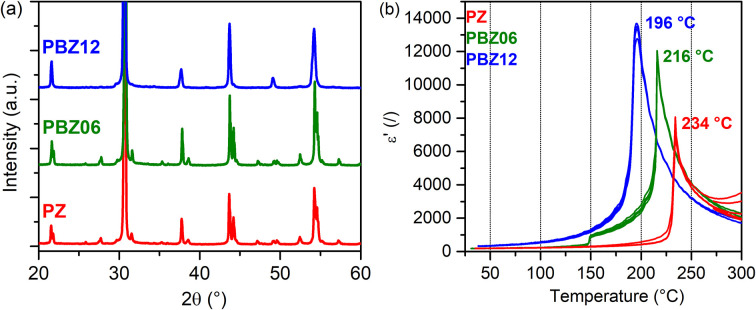
(a) XRD patterns of PZ, PBZ06, and PBZ12. (b) *ε*′(*T*) measurements upon heating for PZ, PBZ06, and PBZ12.

The low-angle segments of the XRD patterns of PbZrO_3_ (PZ), Pb_0.94_Ba_0.06_ZrO_3_ (PBZ06), and Pb_0.88_Ba_0.12_ZrO_3_ (PBZ12) are depicted in Fig. 1a. While the diffractograms of PZ and PBZ06 are qualitatively similar, PBZ12 lacks several superlattice reflections and additional splittings in the range of 20–60°. This difference is confirmed by our Rietveld analysis (Fig. S1†), which allows refinement with orthorhombic (space group no. 55, *Pbam*) symmetry for PZ and PBZ06, but indicates a predominantly rhombohedral (no. 161, *R*3*c*) symmetry in the case of PBZ12, in accordance with previously published data.^27^ This confirms the composition-induced AFE → FE phase transition when the amount of barium at the perovskite A-site is increased from *x* = 0.06 to 0.12. However, we note that a minor amount of the orthorhombic phase was also detected in PBZ12. In addition, the different states of our XRD samples (ground and annealed powders) in comparison with our NMR samples (sintered pellets) should be kept in mind.

The FE nature of PBZ12 is also supported by the electrical measurements. The polarization–electric field hysteresis loop is shown in the ESI (Fig. S2†). While it has been initially reported that the AFE state in PBZ persists up until *x* = 0.175 at room temperature,^25,26^ the exact location of this phase boundary remains unclear and the transition behavior is complex.^8^ Other experimental studies have reported FE-like polarization hysteresis for *x* = 0.1 and more.^22^ Measurements of the dielectric permittivity *ε*′ as a function of temperature (Fig. 1b) can provide additional information on the phase transitions between the paraelectric (PE), FE, and AFE states. Upon heating, PBZ06 exhibits two anomalies in *ε*′ at around 150 °C and at 216 °C. They are related to two temperature-induced phase transitions and attributed to the AFE → FE and FE → PE transitions, respectively. While the AFE → FE transition is subject to a large thermal hysteresis of around 50 °C, which is expected for a first-order transition,^45,46^ the FE → PE transition has a small hysteresis of 8 K. This behavior is similar to that of pure PZ, although here the first anomaly is only observable upon cooling (Fig. S3†). In contrast, PBZ12 exhibits only a single transition at 196 °C (heating), further supporting our conclusion on its room-temperature FE state.

**Fig. 2:**
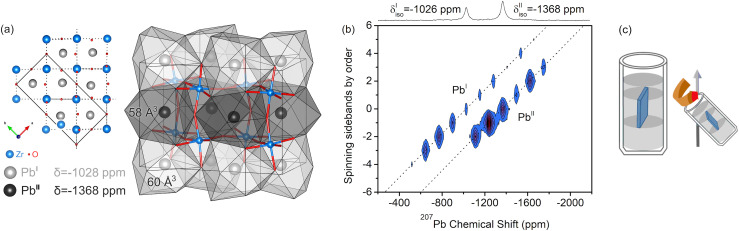
(a) Structure of orthorhombic (*Pbam*) PZ^9^ with crystallographic sites Pb^I^ and Pb^II^ visualized with the program VESTA.^44^ The two sites feature differently sized coordination spheres and different displacement magnitudes. In ^207^Pb NMR spectra, the site with the shortest Pb–O distance is assigned to the signal with the less negative isotropic CS. (b) ^207^Pb 2D-PASS spectrum of ceramic PZ and visualization of the sample geometry. The two diagonals correspond to the isotropic signals with SSB manifolds of Pb^I^ and Pb^II^. After a shearing operation, the purely isotropic signal displayed in the top projection can be obtained.

The structure of orthorhombic (*Pbam*) PZ^9^ with the crystallographic sites Pb^I^ and Pb^II^ is depicted in Fig. 2a. Its ^207^Pb NMR spectrum at room temperature is known to exhibit two signals corresponding to the two inequivalent sites,^47^ the full CSA tensors of which have been determined from powder line shapes.^48^ Pb^I^ and Pb^II^ differ not only with respect to the volume of the PbO_12_ coordination polyhedra, but also their displacements from the centers thereof which are characteristic of the AFE state.

As shown in the inlay, both sites feature an antiparallel arrangement of displacements. However, the displacement magnitudes vary between the two sites. In NMR, the signal with the less negative isotropic CS and larger CSA has previously been assigned to the lead site with the shortest Pb–O distance.^35,39^ With the 2D-PASS pulse sequence (Fig. 2b), the two signals can be separated along the diagonals of the spectrum, and simulations of the intensity patterns of the extracted SSB manifolds yield *δ*^Pb^I^^ = −1026 ppm, CSA^Pb^I^^ = −865 ± 20 ppm and *δ*^Pb^II^^ = −1368 ppm, CSA^Pb^II^^ = −550 ± 10 ppm. The NMR parameters are qualitatively in line with the previously reported values for PZ (Table S1†), and we suggest that any deviations of our data can be attributed to temperature gradients in the sample configuration, which can result in frictional heating.^49^ At the same time, we have not observed significant differences in line shape between pellet and powder samples (Fig. S4†). The chemical shift of PZ has been reported to be temperature-dependent between 0 and 30 °C,^39^ which is why we refrain from assigning too much importance to minor chemical shift changes in this work.

**Fig. 3:**
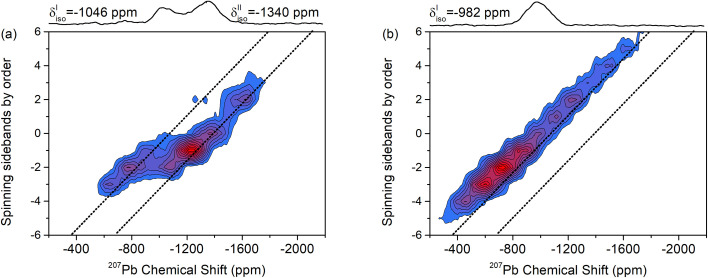
(a) ^207^Pb 2D-PASS spectra and sum projections after shear (purely isotropic projections) of (a) a PBZ06 pellet and (b) a PBZ12 pellet recorded at 8 kHz MAS. The diagonal positions of the two sites in PZ have been included as a guide to the eye.

The 2D-PASS spectrum of PBZ06 depicted in Fig. 3a still features two distinct sites but is broadened compared to the spectrum of pure PZ. In a Hahn-Echo spectrum of the same ceramic pellet, this additional line width is significant enough to prevent any meaningful extraction of NMR parameters due to the heavy overlap of broadened SSB (Fig. S5†). 2D-PASS, in contrast, still allows for the discrimination between the two SSB manifolds. After shearing the individual rows of the spectrum with multiples of the MAS rate (Fig. S6†), a sum projection of the rows only consists of the isotropic contributions to the CS.^41,47^ As shown in Fig. 3a, this “purely isotropic” projection features two distinct signals with maxima at *δ*^Pb^I^^ = −1025 ppm and *δ*^Pb^II^^ = −1355 ppm for PBZ06.

**Fig. 4:**
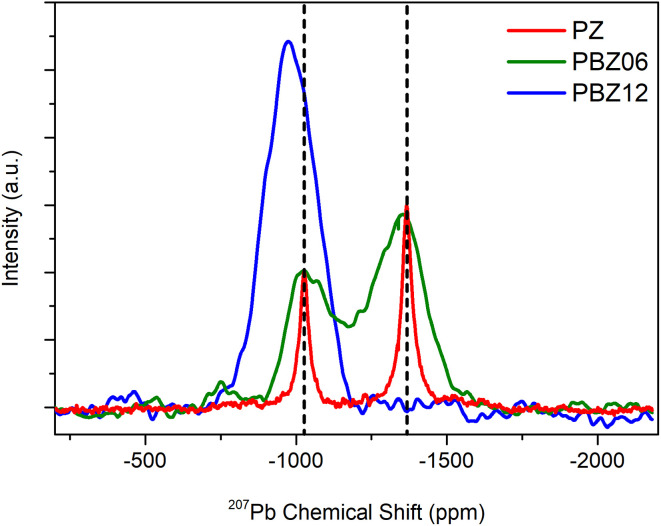
Comparison of purely isotropic projections taken from the ^207^Pb 2D-PASS spectra of the ceramic pellets of PZ, PBZ06, and PBZ12 shown in Fig. 2 and 3.

A superposition of the purely isotropic projections is depicted in Fig. 4. The increase of Gaussian line width between PZ and PBZ06 from *Δ*^Pb^I^^ = 39 ppm and *Δ*^Pb^II^^ = 45 ppm to *Δ*^Pb^I^^ = 187 ppm and *Δ*^Pb^II^^ = 217 ppm (Fig. S7 and Table S2†) is due to a dispersion of isotropic CS. This indicates the presence of a distribution of varying local environments, each of which contributes a slightly different CS value to the signal. The FWHM of the purely isotropic NMR lines is thus a measure of the underlying parameter distributions. We suggest that, in principle, two different effects might cause the increase of the width of the chemical shift distributions upon the addition of barium. Firstly, the presence of next-nearest neighboring (NNN) barium atoms could cause binary changes in the direct local environment of surrounding Pb^2+^ cations (compositional disorder). Secondly, the addition of barium to the lattice could cause continuous variations in the Pb–O bond length distributions (displacive disorder).

Assuming a random distribution of A-site cations, the probability of a Pb^2+^ cation in PBZ06 to have at least one next-nearest Ba^2+^ neighbor is 31% (Fig. S8†). Hence, any compositional contribution to the disorder would only be attributed to a small fraction of Pb^2+^ cations. In addition, such an effect is expected to result in a bimodal line shape reflecting the presence of two distinct lead environments for every crystallographic lead site in the structure, which is not supported by our experiments.

On the other hand, Zhou *et al.*^36^ have previously correlated the isotropic CS of ^207^Pb with effective Pb–O bond lengths: when Pb^2+^ cations are displaced from the center of their coordination spheres, the Pb–O distances split up, causing the CS to reflect a bond length that is dominated by the shortest value. In the same sense, an observed increase of line width can be attributed to a significant increase of Pb–O bond length disorder.^34^ They were able to demonstrate that a unimodal Gaussian-like line shape like the one observed in our work could thus be well explained by displacive disorder in a single direction, *i.e.* disorder of displacement magnitudes. Thus, we argue here that the distribution of isotropic CS observed in barium-modified PZ is mainly due to this kind of displacive disorder; however, we cannot exclude a combined effect.

Applying the relationships established by Fayon *et al.*,^36,37^ we can estimate that the distributions of effective bond lengths increase from *σ*(Pb^I^–O) = *σ*(Pb^II^–O) = 0.002 Å for PZ to *σ*(Pb^I^–O) = 0.009 Å and *σ*(Pb^II^–O) = 0.011 Å for PBZ06. Thus, it increases by one order of magnitude as a result of the addition of 6% barium. The relative peak areas of the two signals are found to be 38% and 62% in PBZ06 compared to 37% and 63% in pure PZ. It should be noted that in both cases, they deviate from the expected 1 : 1 intensity ratio. We attribute this to the fact that the r.f. pulse was in resonance with the Pb^II^ signal and therefore had a higher excitation efficiency for this signal.

The spectrum of PBZ12 only exhibits one diagonal (Fig. 3b) corresponding to one broad signal with a maximum at −975 ppm in the purely isotropic projection (Fig. 4). The disappearance of one signal between PBZ06 and PBZ12 is also evident in the superposition in Fig. 4. In the context of our XRD and dielectric measurements, we can conclude that while the AFE state of PBZ features the two lead sites known from PZ, the FE state has only one distinct site. This result is consistent with the FE state of PBZ being an *R*3*c* perovskite with only one crystallographic A-site (Fig. S9†).^50^ Considering the relationship between lead displacements and effective Pb–O bond lengths, we can also interpret the disappearance of one signal as the collapse of two different displacement magnitudes into one.

**Fig. 5:**
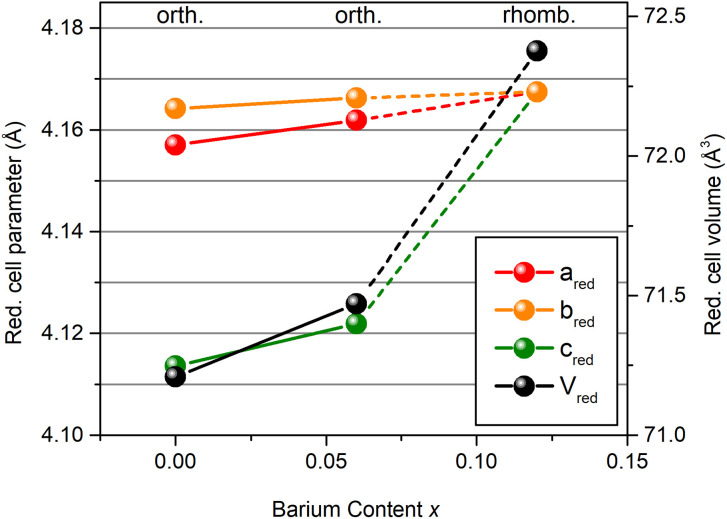
Reduced cell parameters and cell volumes obtained from Rietveld refinements of the XRD data.

The site that prevails in PBZ12 is the one with the more positive isotropic CS and larger CSA. Hence, the coordination sphere and the magnitude of lead displacements in PBZ12 are similar to that of the Pb^I^ site in PZ and PBZ06. Pb^I^ features larger displacements and the shorter shortest Pb–O distance, but longer average bonds. As depicted in Fig. 5, our Rietveld refinements find that the lattice volume of the reduced perovskite unit cell significantly increases during the AFE → FE transition from 71.47 Å^3^ (PBZ06) to 72.38 Å^3^ (PBZ12). This is mostly due to an abrupt increase in the reduced *c* parameter. A lattice volume increase with barium content is well in line with the larger ionic radii (*r*(Ba^2+^) = 1.61 Å, *r*(Pb^2+^) = 1.49 Å),^51^ and has also been reported by Pokharel *et al.*^46^ We suggest that the global lattice expansion translates into lattice expansions around the barium atoms on a local scale, which is in agreement with a larger, Pb^I^-like coordination sphere being favored by the local structure. At the same time, the Pb^I^ sites also feature larger lead displacements, indicating that the lead ions prefer off-center positions as the cell expands. A similar effect has been reported by Zhou *et al.*^36^ for (1 − *x*)PMN-*x*PSN solid solutions, albeit for B-site substitution. The resemblance of the local lead environment in PBZ12 to the Pb^I^ site in PZ and PBZ06 can invite speculation on whether their local displacement directions are the same despite their long-range structures indicating average displacements along different directions. However, the Gaussian-style broadening of the NMR signals only allows us to infer that displacements occur in a single, unique direction^33,36^ which may differ from the average polarization direction.^19,52^ For more in-depth conclusions, a study of the local structure dynamics would be required.

Analyzing the spectra in more detail, slight differences with respect to the spectral parameters can be observed. The line width increases from 188 ppm to 201 ppm between PBZ06 and PBZ12, indicating a further increase of the Pb–O bond length disorder and corresponding to an effective distribution of *σ*(Pb^I^–O) = 0.010 Å. This change is small compared to the strong increase from PZ to PBZ06. Since in PBZ12, the probability of any Pb^2+^ cation to have at least one Ba^2+^ NNN can be estimated at 55%, part of the increase of the line width from PBZ06 to PBZ12 can in fact be attributed to an increase in compositional disorder.

The isotropic chemical shift of the Pb^I^ site increases by 64 ppm from PBZ06 to PBZ12, reflecting a shortening of effective Pb^I^–O distances. This can be well explained by lead ions moving further off-center in the larger PbO_12_ cavities of the FE structure. It is known from the XRD data that the Pb^I^ site in pure PZ favors larger displacements,^9^ and previous studies have shown that Pb^2+^ cations tend to move off-center from high-symmetry positions to accommodate their lone pair, which can help explain this observation.^52^ Applying the formula by Fayon *et al.*,^37^ a decrease of effective Pb^I^–O distances of roughly 0.007 Å can be estimated. In comparison, the difference between Pb^I^ and Pb^II^ in PZ is 0.03 Å for the average Pb–O distances and 0.04 Å for the shortest distances.^9^ Thus, the change induced in PBZ12 is not on the same order of magnitude as the difference between the two sites.

A possible explanation for the observed local structural changes can be found in the cubic structure and larger lattice volume of BaZrO_3_ compared to PZ.^9,53^ As mentioned above, introducing barium to the lattice might cause a local-scale expansion around the Ba^2+^ cations, facilitating larger Pb^2+^ displacements. As more barium is added to the lattice, these displacements are ultimately correlated as ferroelectricity during the structure-induced AFE → FE phase transition. The experimental data based on the ^207^Pb NMR spectra clearly demonstrate a larger distribution of the shortest Pb–O bond lengths with increasing barium content and a more covalent Pb environment in the barium-rich FE phase. However, the origin of the observed effect, explained in terms of the larger size and lower polarizability of Ba^2+^, and how this affects the local environment of lead sites in the direct neighbourhood of substituents and beyond, remains an open hypothesis to be tested in future studies.

**Fig. 6:**
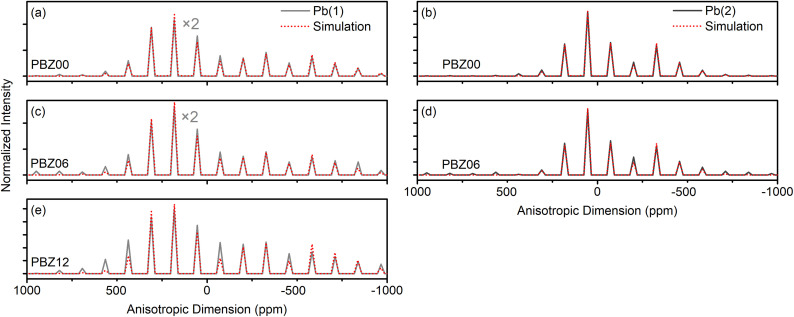
Comparison of the anisotropic projections taken from the ^207^Pb 2D-PASS spectra of PZ, PBZ06, and PBZ12 shown in Fig. 2 and 3. In (a) and (c), the intensity scale is halved for better visibility.

In addition, the different polarizabilities of barium compared to lead^54^ might influence the bonding interactions, a change which should reflect in the correlation of isotropic CS and CSA.^36^ The 2D-PASS spectra contain information about both the isotropic CS and the CSA. Anisotropic projections (Fig. 6) can be obtained as vertical slices at the positions of the signal maxima in the sheared data. While the slices are essentially point spectra and therefore cannot be equated with full experimental SSB manifolds, their widths and intensity patterns are still characteristic of the value of the CSA and can be reproduced with a fit. However, the determination of an accurate value of the asymmetry parameter *η* exceeds the estimated accuracy of the line shape simulation. As the Pb^2+^ cations have their greatest displacements along one axis of symmetry (〈110〉_PC_ in the orthorhombic structure^9^ and 〈111〉_PC_ in the rhombohedral structure^50^), axial symmetry with an asymmetry parameter of *η* ≈ 0 is assumed instead.

Between PZ and PBZ06, no changes of the CSA outside of the estimated margins of error are determined. The chemical shift tensor values fall in the order of magnitude described by Zhou *et al.*^36^ as midway between covalent and ionic. However, for PBZ12, the CSA increases by 50 ppm (Table 1). While the increase is small compared to the estimated maximal margins of error of ≤30 ppm, the increase of SSB intensity between 500 and −500 ppm is still evident in the intensity patterns. It is possible that this change, in combination with the increase of the isotropic CS, reflects a transition to a slightly more covalent/less ionic bonding environment.^36^ This contrasts the notion that Pb-based compositions tend to be more covalent than Ba-based compositions,^55^*i.e.* the Pb^2+^ environment becomes more covalent instead of being less covalent after Ba^2+^ addition. One can speculate that the lower polarizability of Ba^2+^ compared to that of Pb^2+^ could potentially lead to weaker Ba–O bonds, in turn facilitating the formation of stronger Pb–O bonds in their vicinity. Further information on the structural changes could be obtained in the future by neutron diffraction, EXAFS or HRTEM experiments, which are beyond the scope of the current study.

**Table 1:** Isotropic CS taken from the signal maxima and corresponding values of the CSA determined from the fits shown in Fig. 6. Margins of error of the isotropic CS are reading errors and are higher for PBZ06 due to signal overlap. Maximal margins of errors of the CSA are estimated based on visual judgments of the fits after manually varying the CSA, as recalled in the ESI†

	Pb^I^		Pb^II^	
	*δ*_iso_/ppm	CSA/ppm	*δ*_iso_/ppm	CSA/ppm
PZ	−1026 ± 2	−865 ± 20	−1368 ± 2	−550 ± 10
PBZ06	−1025 ± 10	−858 ± 18	−1355 ± 10	−510 ± 15
PBZ12	−975 ± 5	−905 ± 30		

After applying an electric field to the PBZ12 sample, the spectrum remains unchanged (Fig. S9 and S10†), further corroborating the interpretation that it is already in the FE state. The lack of changes in the NMR spectra indicates that unpoled and poled FE states for this compound merely differ by microstructural texturing of the sample. Studying PZ and PBZ06 post-electric fields by ^207^Pb NMR spectroscopy could give insights into the reversibility of the AFE → FE transitions in these compositions. However, the electric fields necessary to induce the phase transitions exceeded the dielectric breakdown strengths at room temperature in the case of our samples, rendering such analysis inaccessible in our study.

## Conclusions

In this work, changes in the local environment of lead upon substitution with barium were analyzed in the AFE model system (Pb_1−*x*_Ba_*x*_)ZrO_3_ by ^207^Pb NMR spectroscopy. ^207^Pb isotropic chemical shifts reflect effective Pb–O distances and therefore, displacements of Pb^2+^ ions within their oxygen cages. The electric field-induced AFE → FE phase transition in ceramic PbZrO_3_ is irreversible; for *x* = 0.06, the AFE state is expected to be destabilized. ^207^Pb NMR revealed an increase of isotropic line widths, indicating that the substitution with barium induces Pb–O bond length disorder with a distribution width of approx. 0.01 Å. Meanwhile, the isotropic chemical shift, the relative integrals of the Pb^I^:Pb^II^ signals, and the CSA remained unchanged between the two compounds, reflecting that the global structure is not strongly influenced by the introduction of barium.

Upon crossing a threshold value for *x*, barium induces a macroscopic phase transition to a FE state, which was observed by XRD and, on a local scale, the collapse of two lead environments into one. For *x* = 0.12, lead environments and displacements are of similar nature to those of the Pb^I^ site in pure PZ. The stabilization of the larger volume site is in accordance with a lattice expansion as determined by Rietveld refinements. Meanwhile, the favoring of shorter shortest Pb–O distances reflects a tendency for larger Pb^2+^ displacements. We suggest that the introduction of Ba^2+^ ions to the lattice can cause local-scale lattice expansions in their vicinity, facilitating the displacements. Compared to the Pb^I^ site in PbZrO_3_, a further off-center shift of Pb^2+^ ions in the larger PbO_12_ cavities of the FE structure is observed. An accompanying increase of the CSA gives an indication of a more covalent bonding environment in PBZ12, which could potentially be related to the lower polarizability of Ba^2+^ facilitating the formation of stronger Pb–O bonds in their vicinity. From the local structural point of view, the composition-induced AFE → FE phase transition can therefore potentially be described as an increasing correlation of larger lead displacements in larger oxygen cavities as more barium is added to the lattice.

The dielectric breakdown did not allow for an *ex situ* study of the (Pb_1−*x*_Ba_*x*_)ZrO_3_ post-electric field for *x* = 0, 0.06 in this study. For *x* = 0.12, no changes were observed (Fig. S10 and 11†), indicating that the poled ferroelectric state for this compound only differs with respect to the microstructural texturing of the sample.

## Conflicts of interest

There are no conflicts to declare.

## Supplementary Material

DT-051-D2DT01302A-s001

## References

[cit1] Liu Z., Lu T., Ye J., Wang G., Dong X., Withers R., Liu Y. (2018). Antiferroelectrics for Energy Storage Applications: A Review. Adv. Mater. Technol..

[cit2] Mischenko A., Zhang Q., Scott J. F., Whatmore R. W., Mathur N. D. (2006). Giant Electrocaloric Effect in Thin Film PbZr_0.95_Ti_0.05_O_3_. Science.

[cit3] Novak N., Weyland F., Patel S., Guo H., Tan X., Rödel J., Koruza J. (2018). Interplay of Conventional with Inverse Electrocaloric Response in (Pb,Nb)(Zr,Sn,Ti)O_3_ Antiferroelectric Materials. Phys. Rev. B.

[cit4] Sawaguchi E., Shirane G., Takagi Y. (1951). Phase Transition in Lead Zirconate. J. Phys. Soc. Jpn..

[cit5] Shirane G., Sawaguchi E., Takagi Y. (1951). Dielectric Properties of Lead Zirconate. Phys. Rev..

[cit6] Shirane G. (1952). Ferroelectricity and Antiferroelectricity in Ceramic PbZrO_3_ Containing Ba or Sr. Phys. Rev..

[cit7] Chien P.-H., Griffith K. J., Liu H., Gan Z., Hu Y.-Y. (2020). Recent Advances in Solid-State Nuclear Magnetic Resonance Techniques for Materials Research. Annu. Rev. Mater. Res..

[cit8] Liu H., Dkhil B. (2011). A Brief Review on the Model Antiferroelectric PbZrO_3_ Perovskite-Like Material. Z. Kristallogr..

[cit9] Corker D. L., Glazer A. M., Dec J., Roleder K., Whatmore R. W. (1997). A Re-Investigation of the Crystal Structure of the Perovskite PbZrO_3_ by X-ray and Neutron Diffraction. Acta Crystallogr., Sect. B: Struct. Sci..

[cit10] Tan X., Ma C., Frederick J., Beckman S., Webber K. G. (2011). The Antiferroelectric ↔ Ferroelectric Phase Transition in Lead-Containing and Lead-Free Perovskite Ceramics. J. Am. Ceram. Soc..

[cit11] Jona F., Shirane G., Mazzi F., Pepinsky R. (1957). X-Ray and Neutron Diffraction Study of Antiferroelectric Lead Zirconate, PbZrO_3_. Phys. Rev..

[cit12] Sawaguchi E., Maniwa H., Hoshino S. (1951). Antiferroelectric Structure of Lead Zirconate. Phys. Rev..

[cit13] Glazer A. M. (1993). Structure and Disorder in Single-Crystal Lead Zirconate, PbZrO_3_. Acta Crystallogr., Sect. B: Struct. Sci..

[cit14] Xu B., Hellman O., Bellaiche L. (2019). Order-Disorder Transition in the Prototypical Antiferroelectric PbZrO_3_. Phys. Rev. B.

[cit15] Íñiguez J., Stengel M., Prosandeev S., Bellaiche L. (2014). First-Principles Study of the Multimode Antiferroelectric Transition in PbZrO_3_. Phys. Rev. B: Condens. Matter Mater. Phys..

[cit16] Tagantsev A. K., Vaideeswaran K., Vakhrushev S. B., Filimonov A. V., Burkovsky R. G., Shaganov A., Andronikova D., Rudskoy A. I., Baron A. Q. R., Uchiyama H., Chernyshov D., Bosak A., Ujma Z., Roleder K., Majchrowski A., Ko J.-H., Setter N. (2013). The Origin of Antiferroelectricity in PbZrO_3_. Nat. Commun..

[cit17] Vales-Castro P., Roleder K., Zhao L., Li J.-F., Kajewski D., Catalan G. (2018). Flexoelectricity in Antiferroelectrics. Appl. Phys. Lett..

[cit18] Liu H. (2018). Origin of the Intermediate Phase in Lead Zirconate, PbZrO_3_. J. Am. Ceram. Soc..

[cit19] Teslic S., Egami T., Viehland D. (1997). Structural Instabilities in PZT. Ferroelectrics.

[cit20] Fesenko O. E., Kolesova R. V., Sindeyev Y. G. (1978). The Structural Phase Transitions in Lead Zirconate in Super-High Electric Fields. Ferroelectrics.

[cit21] Hao X., Zhai J., Kong L. B., Xu Z. (2014). A Comprehensive Review on the Progress of Lead Zirconate-Based Antiferroelectric Materials. Prog. Mater. Sci..

[cit22] Yoon K. H., Hwang S. C. (1997). Dielectric and Field-Induced Strain Behaviour of (Pb_1−x_Ba_x_)ZrO_3_ Ceramics. J. Mater. Sci..

[cit23] Roberts S. (1950). Dielectric Properties of Lead Zirconate and Barium-Lead Zirconate. J. Am. Ceram. Soc..

[cit24] Shirane G., Hoshino S. (1954). X-Ray Study of Phase Transitions in PbZrO_3_ Containing Ba or Sr. Acta Crystallogr..

[cit25] El-Harrad I., Ridah A., Carabatos-Nédelec C., Becker P., Handerek J., Ujma Z., Dmytrow D. (1998). Raman Scattering Investigation with Temperature of Phase Transitions in (Pb_1−x_Ba_*x*_)ZrO_3_ Ceramics at Critical Compositions x = 0.175 and 0.35. J. Raman Spectrosc..

[cit26] Pokharel B. P., Pandey D. (2002). Effect of Ba^2+^ Substitution on the Stability of the Antiferroelectric and Ferroelectric Phases in (Pb_1−x_Ba_x_)ZrO_3_: Phenomenological Theory Considerations. Phys. Rev. B: Condens. Matter Mater. Phys..

[cit27] Pokharel B. P., Ranjan R., Pandey D., Siruguri V., Paranjpe S. K. (1999). Rhombohedral Superlattice Structure and Relaxor Ferroelectric Behavior of (Pb_0.70_Ba_0.30_)ZrO_3_ Ceramics. Appl. Phys. Lett..

[cit28] Pokharel B. P., Pandey D. (1999). Irreversibility of the Antiferroelectric to Ferroelectric Phase Transition in (Pb_0.90_Ba_0.10_)ZrO_3_ ceramics. J. Appl. Phys..

[cit29] Rödel J., Li J.-F. (2018). Lead-Free Piezoceramics: Status and Perspectives. MRS Bull..

[cit30] Zhang M.-H., Fulanović L., Egert S., Ding H., Groszewicz P. B., Kleebe H.-J., Molina-Luna L., Koruza J. (2020). Electric-Field-Induced Antiferroelectric to Ferroelectric Phase Transition in Polycrystalline NaNbO_3_. Acta Mater..

[cit31] Zhang M.-H., Hadaeghi N., Egert S., Ding H., Zhang H., Groszewicz P. B., Buntkowsky G., Klein A., Koruza J. (2021). Design of Lead-Free Antiferroelectric (1−x)NaNbO_3−x_SrSnO_3_ Compositions Guided by First-Principles Calculations. Chem. Mater..

[cit32] Groszewicz P. B. (2021). NMR Spectroscopy of Electroceramics – Applications to Lead-Free Perovskite Oxides. Open Ceram..

[cit33] Avalos C. E., Walder B. J., Emsley L. (2019). Lead–Oxygen Bond Length Distributions of the Relaxor Ferroelectric 0.67PbMg_1/3_Nb_2/3_O_3–0.33_PbTiO_3_ from ^207^Pb Nuclear Magnetic Resonance. J. Phys. Chem. C.

[cit34] Baldwin A., Thomas P. A., Dupree R. (2005). A Multi-Nuclear NMR Study of the Local Structure of Lead Zirconate Titanate, PbZr_1−x_Ti_*x*_O_3_. J. Phys.: Condens. Matter.

[cit35] Zhao P., Prasad S., Huang J., Fitzgerald J. J., Shore J. S. (1999). Lead-207 NMR Spectroscopic Study of Lead-Based Electronic Materials and Related Lead Oxides. J. Phys. Chem. B.

[cit36] Zhou D. H., Hoatson G. L., Vold R. L., Fayon F. (2004). Local Structure in Perovskite Relaxor Ferroelectrics by ^207^Pb NMR. Phys. Rev. B: Condens. Matter Mater. Phys..

[cit37] Fayon F., Farnan I., Bessada C., Massiot D., Coutures J. P. (1997). Empirical Correlations Between ^207^Pb NMR Chemical Shifts and Structure in Solids. J. Am. Chem. Soc..

[cit38] Dybowski C., Neue G. (2002). Solid State ^207^Pb NMR Spectroscopy. Prog. Nucl. Magn. Reson. Spectrosc..

[cit39] Rossi P., Dvorak M. R., Harbison G. S. (2000). ^207^Pb NMR of PbZrO_3_ and PbZr_1−x_Ti_x_O_3_ Solid Solutions. MRS Online Proc. Libr..

[cit40] Fayon F., Bessada C., Douy A., Massiot D. (1999). Chemical Bonding of Lead in Glasses Through Isotropic vs Anisotropic Correlation: PASS Shifted Echo. J. Magn. Reson..

[cit41] Antzutkin O. N., Shekar S. C., Levitt M. H. (1995). Two-Dimensional Sideband Separation in Magic-Angle-Spinning NMR. J. Magn. Reson., Ser. A.

[cit42] Petříček V., Dušek M., Palatinus L. (2014). Crystallographic Computing System JANA2006: General Features. Z. Kristallogr..

[cit43] Massiot D., Fayon F., Capron M., King I., Le Calvé S., Alonso B., Durand J.-O., Bujoli B., Gan Z., Hoatson G. (2002). Modelling One- and Two-Dimensional Solid-State NMR Spectra. Magn. Reson. Chem..

[cit44] Momma K., Izumi F. (2011). VESTA 3 for Three-Dimensional Visualization of Crystal, Volumetric and Morphology Data. J. Appl. Crystallogr..

[cit45] Pokharel B. P., Pandey D. (2000). Dielectric Studies of Phase Transitions in (Pb_1−x_Ba_x_)ZrO_3_. J. Appl. Phys..

[cit46] Pokharel B. P., Pandey D. (2001). High Temperature X-Ray Diffraction Studies on Antiferroelectric and Ferroelectric Phase Transitions in (Pb_1−x_Ba_x_)ZrO_3_ (x = 0.05, 0.10). J. Appl. Phys..

[cit47] Vogt F. G., Gibson J. M., Aurentz D. J., Mueller K. T., Benesi A. J. (2000). Multiple-Rotor-Cycle 2D PASS Experiments with Applications to ^207^Pb NMR Spectroscopy. J. Magn. Reson..

[cit48] Neue G., Dybowski C., Smith M. L., Hepp M. A., Perry D. L. (1996). Determination of ^207^Pb^2+^ Chemical Shift Tensors from Precise Powder Lineshape Analysis. Solid State Nucl. Magn. Reson..

[cit49] Bielecki A., Burum D. P. (1995). Temperature Dependence of ^207^Pb MAS Spectra of Solid Lead Nitrate. An Accurate, Sensitive Thermometer for Variable-Temperature MAS. J. Magn. Reson., Ser. A.

[cit50] Yokota H., Zhang N., Taylor A. E., Thomas P. A., Glazer A. M. (2009). Crystal Structure of the Rhombohedral Phase of PbZr_1−x_Ti_x_O_3_ Ceramics at Room Temperature. Phys. Rev. B: Condens. Matter Mater. Phys..

[cit51] Shannon R. D. (1976). Revised Effective Ionic Radii and Systematic Studies of Interatomic Distances in Halides and Chalcogenides. Acta Crystallogr., Sect. A: Cryst. Phys., Diffr., Theor. Gen. Crystallogr..

[cit52] Dmowski W., Akbas M. K., Davies P. K., Egami T. (2000). Local Structure of Pb(Sc_1/2_,Ta_1/2_)O_3_ and Related Compounds. J. Phys. Chem. Solids.

[cit53] Perrichon A., Jedvik Granhed E., Romanelli G., Piovano A., Lindman A., Hyldgaard P., Wahnström G., Karlsson M. (2020). Unraveling the Ground-State Structure of BaZrO_3_ by Neutron Scattering Experiments and First-Principles Calculations. Chem. Mater..

[cit54] Schwerdtfeger P., Nagle J. K. (2019). 2018 Table of Static Dipole Polarizabilities of the Neutral Elements in the Periodic Table. Mol. Phys..

[cit55] Wu Z., Krakauer H. (2001). Charge-Transfer Electrostatic Model of Compositional Order in Perovskite Alloys. Phys. Rev. B: Condens. Matter Mater. Phys..

