# Supplementary material for: Fundamental workings of chemical substitution at the A-site of perovskite oxides— a 207Pb NMR study of Ba-substituted PbZrO3
Source: Dalton Trans. 2022 Nov 9;51(46):17827–35. doi: 10.1039/d2dt01302a (PMC9706679; doi:10.1039/d2dt01302a)
Supplement: DT-051-D2DT01302A-s001 [file DT-051-D2DT01302A-s001.pdf]

Electronic Supplementary Material (ESI) for Dalton Transactions.
This journal is © The Royal Society of Chemistry 2022

Supporting Information for

Fundamental workings of chemical substitution to the A-site of perovskite oxides - an ^{207}Pb NMR study of Ba-substituted $PbZrO_3$

Sonja Egert[1], Jurij Koruza[2], Hergen Breitzke[1], Changhao Zhao[3], Barbara Malič[4], Gerd Buntkowsky[1*] and Pedro B. Groszewicz[5*]

[1] Eduard Zintl Institute for Inorganic and Physical Chemistry, Technical University of Darmstadt, Darmstadt 64287, Germany.

[2] Institute for Chemistry and Technology of Materials, Graz University of Technology, Graz 8010, Austria.

[3] Department of Materials and Earth Sciences, Nonmetallic Inorganic Materials, Technical University of Darmstadt, Darmstadt 64287, Germany.

[4] Electronic Ceramics Department, Jožef Stefan Institute, Ljubljana, Slovenia.

[5] Department of Radiation Science and Technology, Delft University of Technology, Delft 2629JB, Netherlands.

* Corresponding Authors: p.groszewicz@tudelft.nl, gerd.buntkowsky@chemie.tu-darmstadt.de

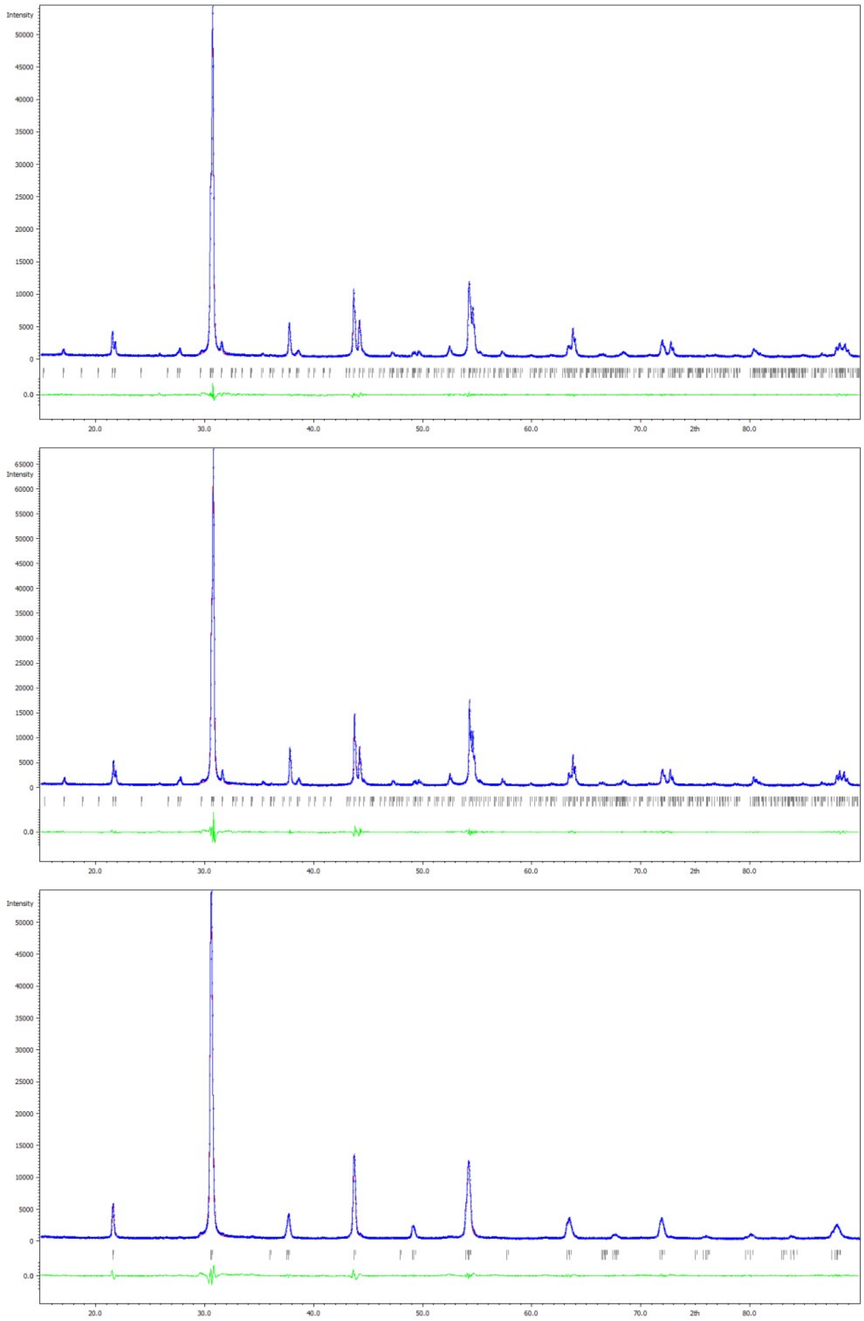

Figure S1: Results of the XRD refinements for the determination of cell parameters for (a) PZ, orthorhombic lattice, (b) PBZ06, orthorhombic, (c) PBZ12, rhombohedral. Blue and red curves represent the measured and calculated data, respectively, while the green line marks the difference curve. Please note that only the background, shift and cell parameters have been refined while the atomic positions were not considered. The refinement was carried out using the Jana2006 software.[1]

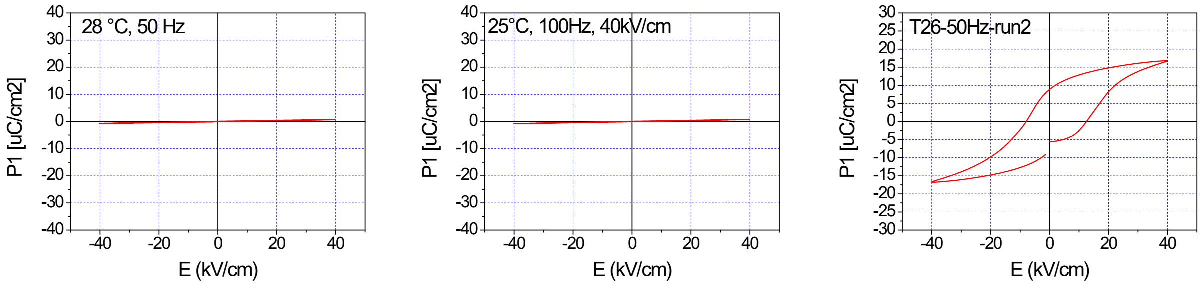

Figure S2: Polarization-electric field hysteresis loops for (a) PZ, (b) PBZ06, (c) PBZ12 at room temperature. Please note that the two AFE compositions PZ and PBZ06 only show linear dielectric behaviour, since at room temperature the dielectric breakdown field is lower than the field needed to induce the AFE-FE phase transition, preventing to obtain the characteristic

double polarization loops.

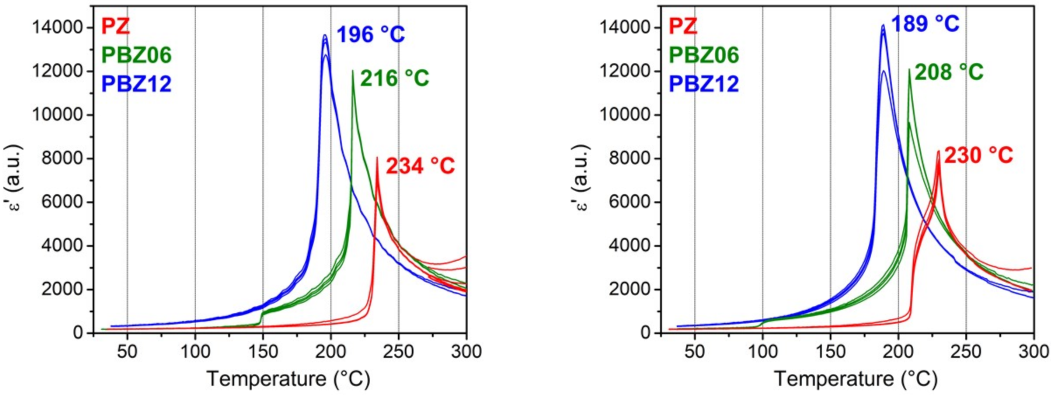

Figure S3: ε(T) measurements (a) upon heating and (b) cooling for PZ, PBZ06, and PBZ12. Heating rate was 2 K/min, while the cooling rate could not be accurately controlled (close to 2 K/min).

Table S1: Selection of previously reported values of ^{207}Pb isotropic CS and CSA (Haeberlen convention) for the two crystallographic sites in $PbZrO_3$.

**δ_{iso} / ppm**		**CSA / ppm**		**η**		**Citation**
Pb^{I}	Pb^{II}	Pb^{I}	Pb^{II}	Pb^{I}	Pb^{II}	
−1000	−1340	-	-	-	-	[2]
−1017	−1363	−838	−546	0.2	0.2	[3]*
−1000	−1349	-	-	-	-	[4]**
−1025±10	−1355±10	-	-	-	-	[5]
−1002	−1366	−973	−562	0.27	0.17	[6]*

*different nomenclature
**different assignment of sites

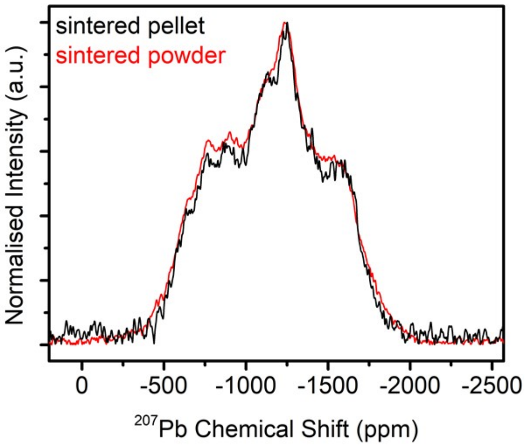

Figure S4: ^{207}Pb Hahn-Echo spectra of PBZ06 as a sintered pellet, and a sintered and annealed powder, at 8 kHz MAS. No significant differences in the line shape are observed.

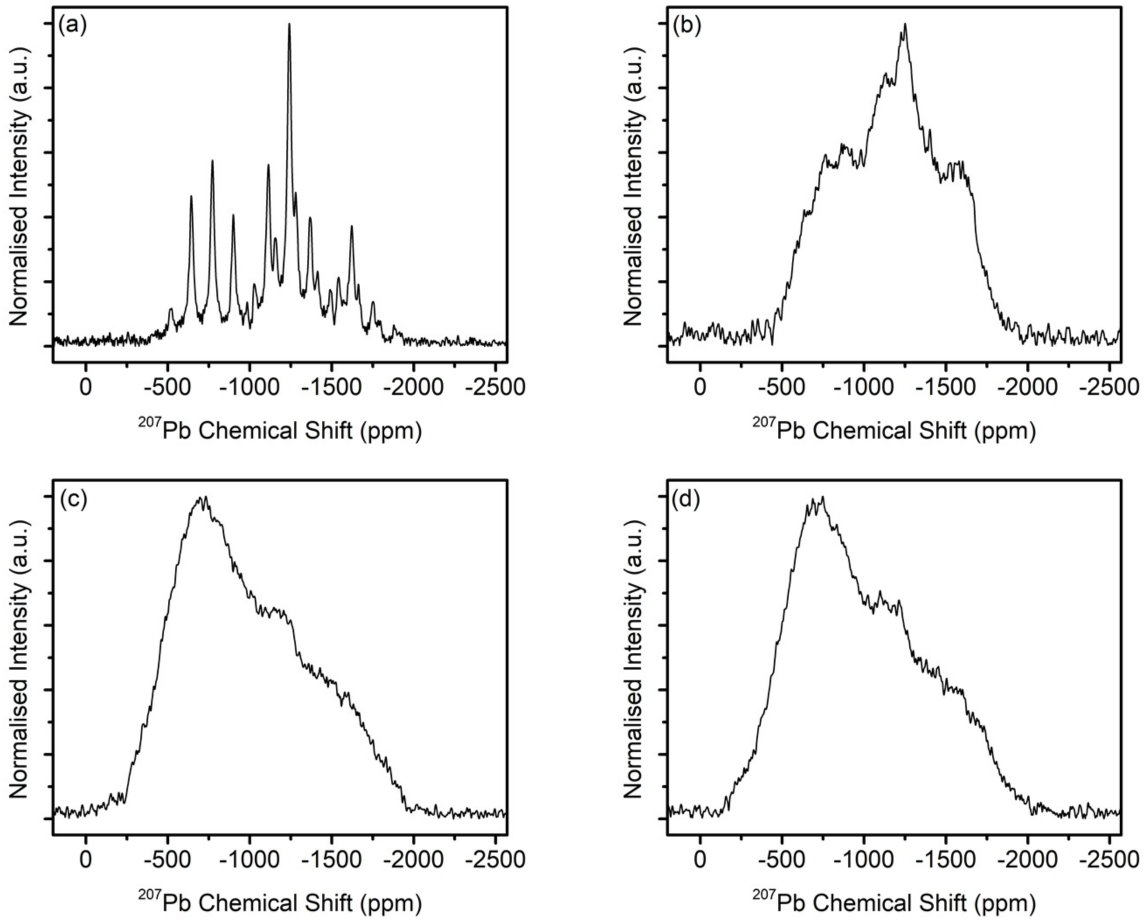

Figure S5: ^{207}Pb Hahn-Echo spectra at 8 kHz MAS of (a) PZ, (b) PBZ06, (c) PBZ12 and (d) PBZ12 after electric field. Acquisition parameters are relaxation delay *d1*=20 s, π/2 pulse length *P1*=4.3 µs, π pulse length *P2*=8.3 µs.

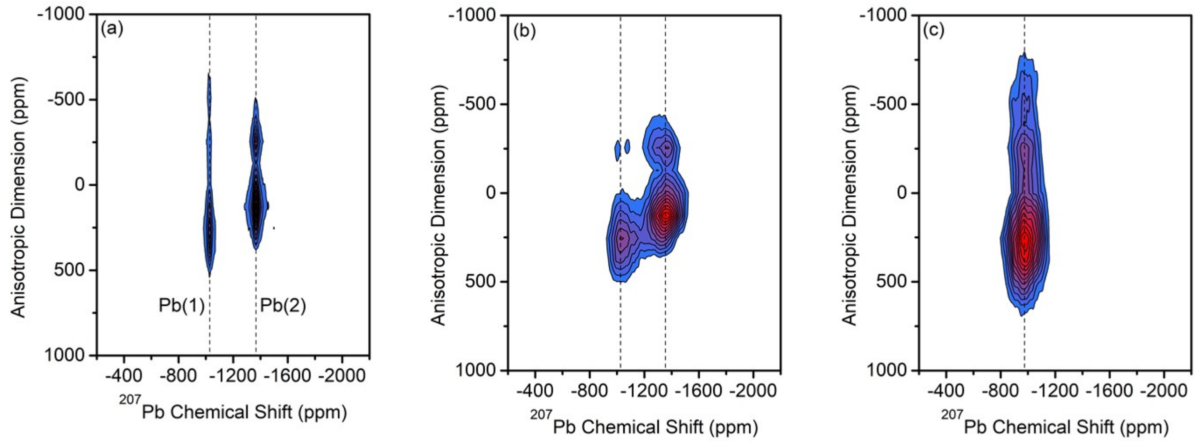

Figure S6: ^{207}Pb 2D-PASS spectra of (a) PZ, (b) PBZ06 and (c) PBZ12 after the F2 shear. The signals of the two crystallographic lead sites are indicated with dashed lines.

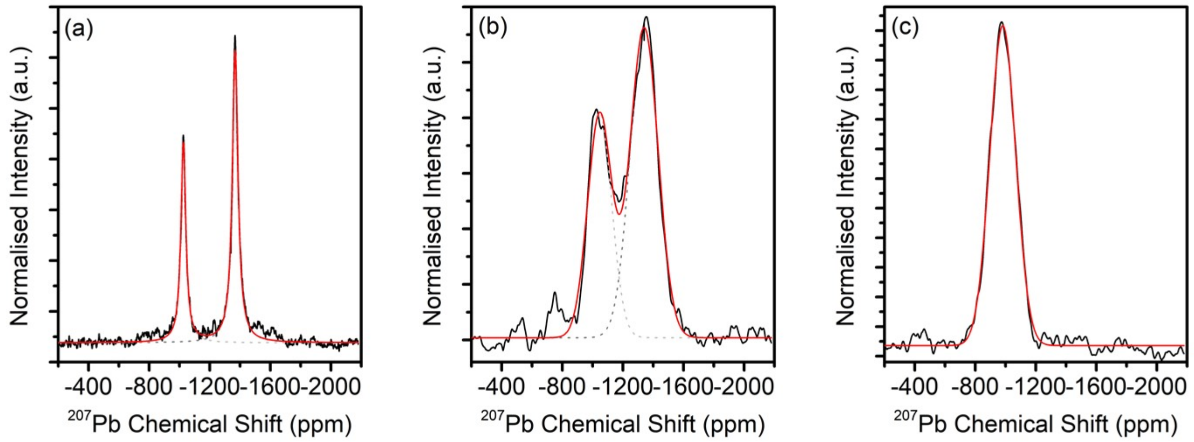

Figure S7: Simulations of the purely isotropic projections of the ^{207}Pb 2D-PASS spectra of (a) PZ with two Lorentzian lines, (b) PBZ06 with two Gaussian lines, and (c) PBZ12 with a single Gaussian.

Table S2: Fit parameters for the isotropic projections shown in Figure S7.

	**Function**	**Position**		**FWHM**		**Area**	
		Pb^I	Pb^{II}	Pb^I	Pb^{II}	Pb^I	Pb^{II}
**PZ**	Lorentzian	−1026	−1368	38.9	45.4	37%	63%
**PBZ06**	Gaussian	−1046	−1340	187.5	217.4	38%	62%
**PBZ12**	Gaussian	−982	-	200.7	-	100%	-

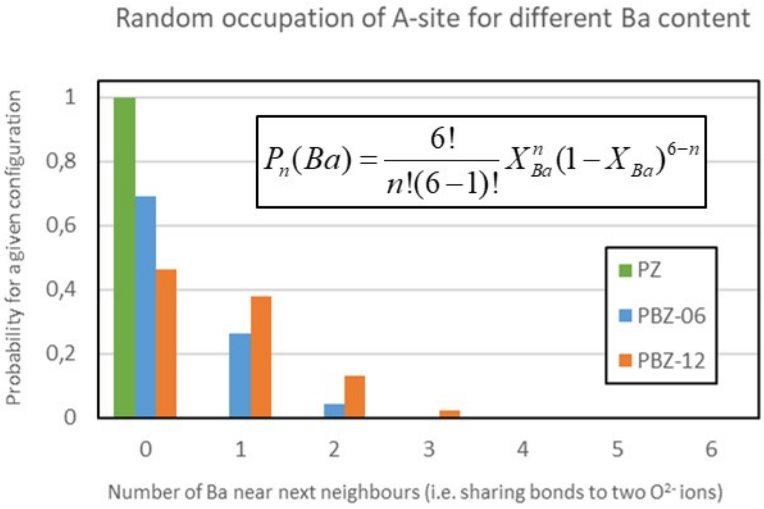

Figure S8: Probability for different numbers of Ba^{2+} cations as next-near neighbours (NNN) of Pb^{2+} sites in PZ-based perovskite oxides. Probabilities are based on a random occupation of the A-site by both cations, as determined from simple statistics given a binomial distribution and the number of possible permutations[7] (see equation in the inset, for which n is the number of Ba^{2+} NNN and X_{Ba} the concentration of Ba^{2+} cations in the solid solution).

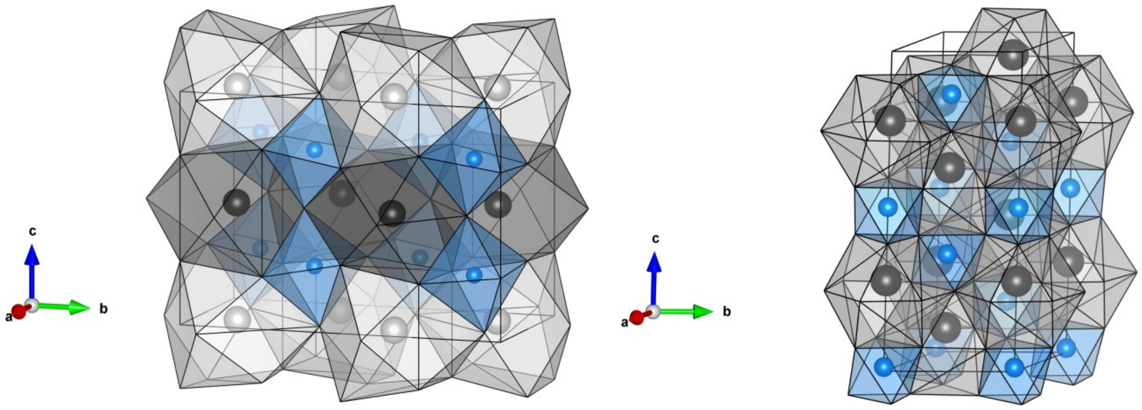

Figure S9: Structural models for (a) nonpolar, AFE, orthorhombic *Pbam* PZ[8] and (b) polar, FE, rhombohedral *R*3*c* $PbZr_{0.9}Ti_{0.1}O_3$.[9] The orthorhombic structure is characterised by two distinct Pb^{2+} sites which each feature antiparallel displacements. The rhombohedral structure is characterised by only one Pb^{2+} site and parallel shifts of the Pb^{2+} ions. Structures were visualized with the VESTA program.[10]

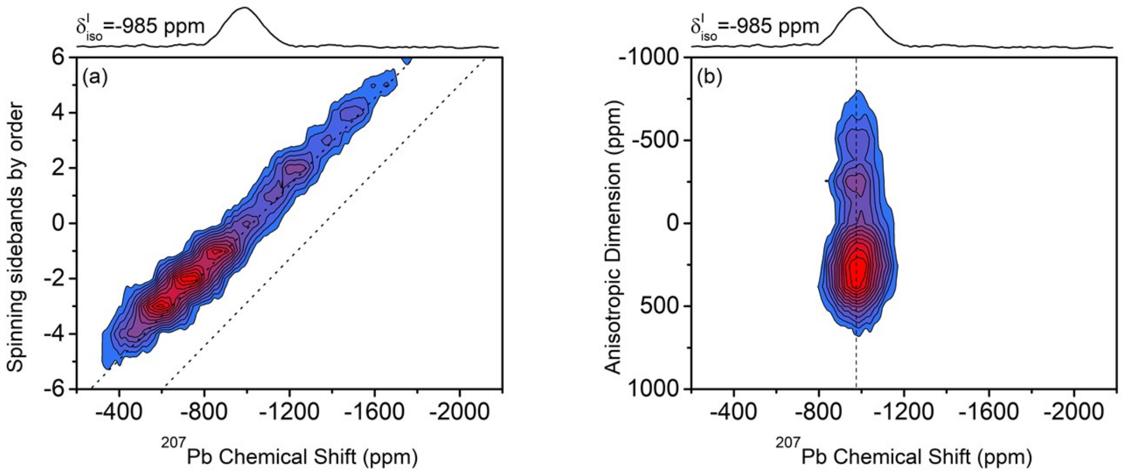

Figure S10: (a) ^{207}Pb 2D-PASS spectrum, sum projection after shear (purely isotropic projection) and (b) sheared 2D-PASS spectrum of a PBZ12 pellet after subjection to an electric field, recorded at 8 kHz MAS.

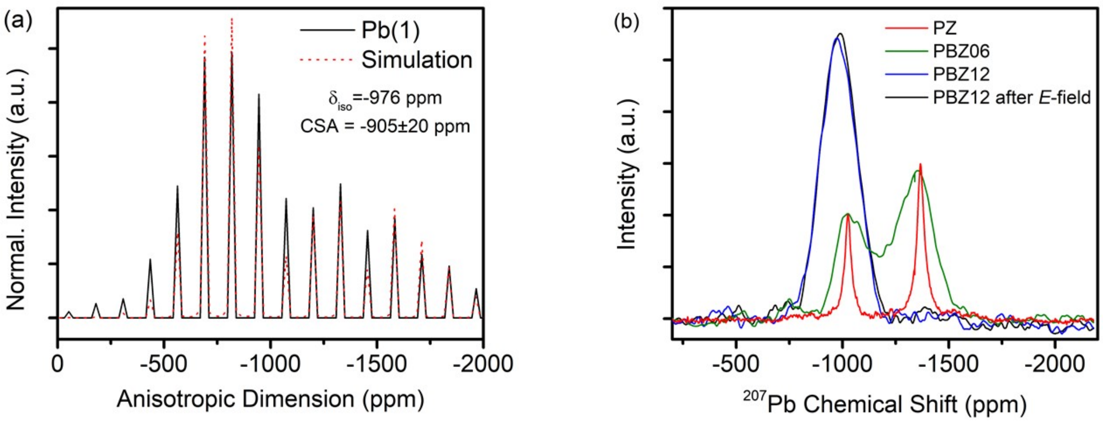

Figure S11: (a) Spinning sideband pattern taken from the ^{207}Pb 2D-PASS spectrum of PBZ12 post-electric field. (b) Comparison of purely isotropic projections taken from the ^{207}Pb 2D-PASS spectra of PZ, PBZ06, PBZ12 and PBZ12 post-electric field.

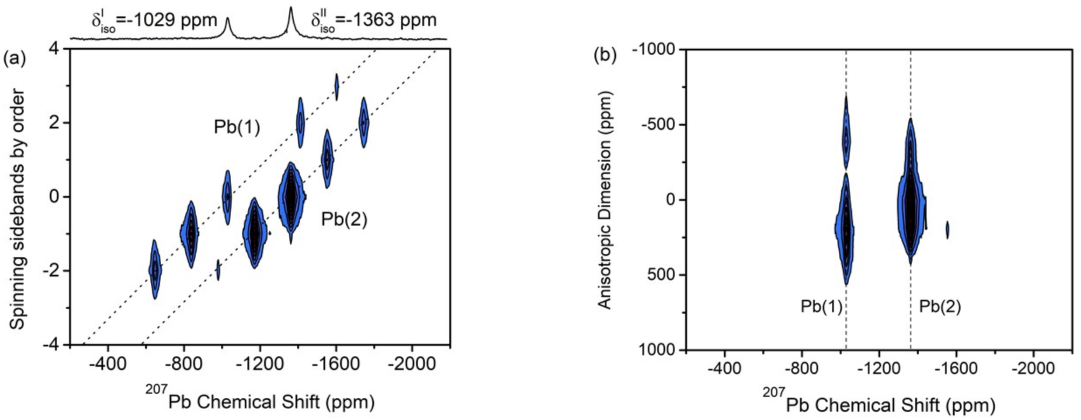

Figure S11: (a) ^{207}Pb 2D-PASS spectrum, sum projection after shear (purely isotropic projection) and (b) sheared 2D-PASS spectrum of commercial $PbZrO_3$ powder. In contrast to the pellet samples, this spectrum was recorded at 12 kHz MAS and with a relaxation delay of *d1*=65 s. The isotropic shift of the Pb^{II} signal is referenced to −1363 ppm, that of the Pb^{I} site is determined to be −1029 ppm.

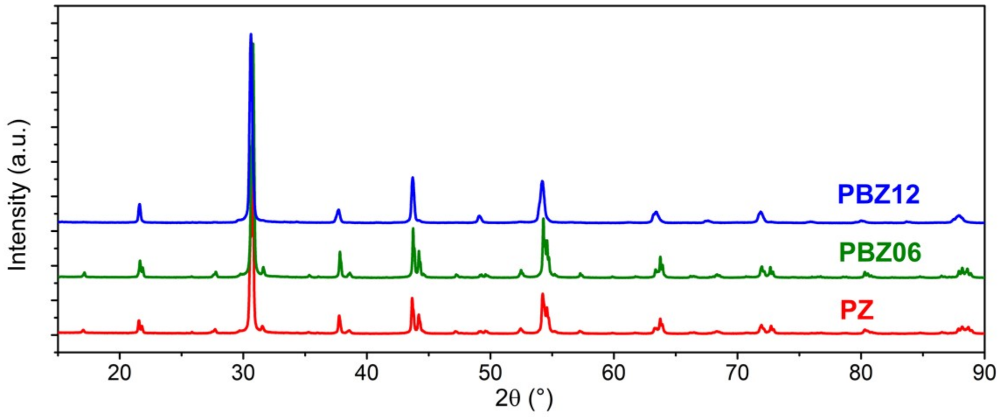

Figure S12: Full XRD patterns of PZ, PBZ06, and PBZ12.

Fits of the anisotropic projections obtained from 2D-PASS spectra

We have estimated the margins of error based on whether a deterioration of the fit quality could be identified by visual judgment. While the errors of the DMFit routine would probably be much smaller in a fully resolved one-dimensional spectrum, we believe this approach to be a reasonable approximation of maximum errors due to the fact that the 2D-PASS slices only contain SSB intensities and no line widths and shapes.

Empirical correlations between NMR and structural parameters

Fayon *et al.*[11] have established empirical correlations between ^{207}Pb isotropic chemical shifts and structural parameters for 21 different lead oxides. The correlation used in our work is, as in Zhou *et al.*[12] and Avalos *et al.*[13]

$$\delta_{iso}(ppm) = 20854 - 8669 r_{Pb-O}(\text{Å}) \qquad (1)$$

The measured isotropic chemical shifts of δ^{PbI} =−1028 ppm and δ^{PbII} =−1368 ppm correspond to Pb-O distances of approximately 2.52 Å and 2.56 Å and are therefore well in accordance with the values of the two shortest distances reported by Glazer *et al.*[14]

We have calculated the distribution width of isotropic chemical shifts from the FWHM according to:

$$FWHM = 2\sigma\sqrt{2\ln 2} \qquad (2)$$

From (1), the distribution width of the isotropic CS is given as:

$$\sigma(Pb-O) = \frac{\sigma(\delta_{iso})}{8669} \qquad (3)$$

Bibliography

[1] V. Petříček, M. Dušek, L. Palatinus, *Z. Kristallogr.* **2014**, *229*, 345.

[2] A. D. Irwin, C. D. Chandler, R. Assink, M. J. Hampden-Smith, *Inorg. Chem.* **1994**, *33*, 1005.

[3] P. Zhao, S. Prasad, J. Huang, J. J. Fitzgerald, J. S. Shore, *J. Phys. Chem. B* **1999**, *103*, 10617.

[4] F. G. Vogt, J. M. Gibson, D. J. Aurentz, K. T. Mueller, A. J. Benesi, *J. Magn. Reson.* **2000**, *143*, 153.

[5] A. Baldwin, P. A. Thomas, R. Dupree, *J. Phys.: Condens. Matter* **2005**, *17*, 7159.

[6] I. Bykov, Y. Zagorodniy, L. Yurchenko, A. Korduban, K. Nejezchleb, V. Trachevsky, V. Dimza, L. Jastrabik, A. Dejneka, *IEEE Trans. Ultrason. Ferroelectr. Freq. Control* **2014**, *61*, 1379.

[7] S.E. Ashbrook, K. R. Whittle, G.R. Lumpkin, I. Farnan, *J. Phys. Chem. B* **2006**, 110, 10358.

[8] D. L. Corker, A. M. Glazer, J. Dec, K. Roleder, R. W. Whatmore, *Acta Cryst.* **1997**, *B53*, 135.

[9] H. Yokota, N. Zhang, A. E. Taylor, P. A. Thomas, A. M. Glazer, *Phys. Rev. B* **2009**, *80*, 104109.

[10] K. Momma, F. Izumi, *J. Appl. Crystallogr.* **2011**, *44*, 1272.

[11] F. Fayon, I. Farnan, C. Bessada, D. Massiot, J. P. Coutures, *J. Amer. Chem. Soc.* **1997**, *119*, 6837.

[12] D. H. Zhou, G. L. Hoatson, R. L. Vold, F. Fayon, *Phys. Rev. B* **2004**, *69*, 305.

[13] C. E. Avalos, B. J. Walder, L. Emsley, *J. Phys. Chem. C* **2019**, *123*, 15744.

[14] D. L. Corker, A. M. Glazer, J. Dec, K. Roleder, R. W. Whatmore, *Acta Cryst. B* **1997**, *53*.
